# Time-Course of the Innate Immune Response of the Terrestrial Crustacean *Porcellio scabe*r After Injection of a Single Dose of Lipopolysaccharide

**DOI:** 10.3389/fimmu.2022.867077

**Published:** 2022-05-03

**Authors:** Andraž Dolar, Anita Jemec Kokalj, Damjana Drobne

**Affiliations:** Department of Biology, Biotechnical Faculty, University of Ljubljana, Ljubljana, Slovenia

**Keywords:** *Porcellio scaber*, lipopolysaccharide, immune challenge, time-dependent response, innate immune system

## Abstract

Invertebrates, including crustaceans, rely on cellular and humoral immune responses to protect against extrinsic and intrinsic factors that threaten their integrity. Recently, different immune parameters have been increasingly used as biomarkers of effects of pollutants and environmental change. Here, we describe the dynamics of the innate immune response of the terrestrial crustacean *Porcellio scaber* to injection of a single dose of lipopolysaccharide (LPS), an important molecular surface component of the outer membrane of Gram-negative bacteria. The aim was to provide a basis for interpretation of change in immune parameters as a result of different challenges, including microplastics and nanoplastics exposure. Changes in total and differential numbers of hemocytes, hemocyte viability, and humoral immune parameters (i.e., phenoloxidase-like activity, nitric oxide levels) were assessed at different times (3, 6, 12, 24, 48 h). An injection of 0.5 μg/μL LPS into the body of *P. scaber* resulted in a rapid decrease (3 h after LPS injection) in the total number of hemocytes and reduced viability of the hemocytes. This was accompanied by changed proportions of the different hemocyte types, as a decrease in the numbers of semigranulocytes and granulocytes, and a marked increase in the numbers of hyalinocytes. In addition, phenoloxidase-like activity and nitric oxide levels in the hemolymph were increased at 3 h and 6 h, respectively, after the LPS challenge. Forty-eight hours after LPS injection, the immune parameters in the hemolymph of *P. scaber* had returned to those before the LPS challenge. This suggests that the innate immune system successfully protected *P. scaber* from the deleterious effects of the LPS challenge. These data indicate the need to consider the dynamics of innate immune responses of *P. scaber* when effects of infections, pollutants, or environmental changes are studied. We also propose an approach to test the immunocompetence of organisms after different challenges in ecotoxicity studies, based on the dynamics of their immune responses.

## Introduction

The innate immune system of invertebrates, including crustaceans, consists of cellular and humoral components, while acquired immunity as known of vertebrates did not develop in invertebrates (1). Appropriate activation of the immune system is essential in all organisms to maintain tissue integrity and facilitate the return to homeostasis. The immune response is balanced between the advantage of a rapid and robust ability to deal with internal and external challenges, and the side-effects of an excessively prolonged and energetically costly immune activation (2, 3). In this regard, the dynamics of the immune response as a function of the dose of activating stimuli has been studied in many organisms, but without particular emphasis on temporal resolution.

Time-courses of immune responses can help to distinguish between transient and sustained responses to any given immune challenge. In a transient immune response, the full functions of the organism return over time to those prior to the stimulus, while for more sustained immune responses, they remain at a different level. Therefore, temporal profiling of the immune response can be used to determine the severity of an immune challenge. On this basis, many cellular and humoral immune components (i.e., the immune parameters) in the hemolymph or other tissues of invertebrates, e.g., crustaceans (e.g., muscle, gills, digestive glands) have become important as ecotoxicological biomarkers (4–8). These immune components are commonly used as biomarkers of the health of an organism or to assess its physiological status after exposure to different stressors, such as microbial infections, molting, and nutrition (5–7, 9). Since hemocytes play a crucial role in crustacean immune defense, the total number of freely circulating hemocytes in the hemolymph (i.e., the total hemocyte count; THC), the proportions of the three hemocyte types (i.e., the differential hemocyte count; DHC), and the hemocyte viability represent the three most commonly studied cellular immune parameters (5, 7, 8, 10). On the other hand, antimicrobial peptides, lysozyme and phenoloxidase-like activities, and nitric oxide (NO) levels are also known to be frequently assessed humoral parameters (5, 6, 10). Overall, while the value of immunological biomarkers is in their sensitivity to environmental threats, however their importance lies in indicating effects at higher levels of biological organization.

Changes in commonly measured immunological biomarkers have been routinely studied in crustaceans to investigate the innate immune responses following a variety of immune challenges. These challenges have included: (a) injection with live microorganisms (11–13) or microbial components, i.e., lipopolysaccharides (LPS), laminarins, and β-glucans (14–16); (b) nylon filament insertion (17, 18); and (c) exposure to different environmental stressors, such as temperature, hypoxia, microplastics, nanoparticles, and pesticides (7, 19–21). In particular, studies on crustaceans stimulated with LPS have shown strong immunomodulatory changes (i.e., changes in immune-related parameters), indicating an induction of the immune system as a result of cytotoxicity of LPS (12, 15, 16, 22). LPS-induced effects reported in most studies include a decrease in THC (12, 16), changes in DHC (12, 15), increased production of reactive oxygen species (ROS) and reactive nitrogen species (RNS; e.g., NO) (22–24), and decreased hemocyte viability (16, 20). In addition, LPS-induced degranulation and release of components of the prophenoloxidase (proPO) system from hemocytes have also been reported, which leads to increased phenoloxidase activity (16, 25). In general, many studies suggest that the innate immune system is rapidly activated after immune challenges (e.g., LPS), with dynamic changes in immune component levels that then gradually return to baseline levels (12, 14, 26). Given the results of these studies, understanding the time-courses of immune responses can explain the degree of stress caused by such infections, pollutants, or environmental change, and avoid bias in interpreting the results of ecotoxicity or ecophysiology studies.

The model organism in the present study was the terrestrial crustacean *Porcellio scaber* (Crustacea: Isopoda), which has an essential ecosystem function in terms of litter decomposition, and is recognized as an important test species in environmental studies (27). The dynamics of the innate immune response in the case of stimulated microbial infection has not yet been studied in *P. scaber*, whereas some major components of the immune system have been investigated in previous studies (6, 28). For example, in the hemolymph of *P. scaber*, there are three main types of hemocytes; semigranulocytes, granulocytes, and hyalinocytes (6), which are responsible for various evolutionarily conserved defense mechanisms (28–30). Among the hemocytes, semigranulocytes represent the most abundant type in the hemolymph (~65% of all free cells), and are responsible for nodulation/encapsulation, and to some extent, for phagocytosis (28, 30) and the production of humoral components, such as the proPO-activating system (31). Granulocytes are another type of hemocyte (~17%), and these have granules in the cytoplasm that are more abundant than seen for semigranulocytes (6). The main role of granulocytes is the synthesis of humoral components, such as antimicrobial peptides, clotting factors, and enzymes involved in the proPO cascade (32–34). These humoral components are responsible for the basic humoral defense mechanisms, such as melanization, cytotoxic reactions, and coagulation (35). The third and final type of hemocytes is the agranular hyalinocytes, the smallest cell type (6), which have a phagocytic activity (30, 36).

The aim of the present study was to investigate the time-course of the innate immune response of the terrestrial crustacean *P. scaber* when challenged with LPS. We hypothesized that the levels of the cellular immune parameters measured (i.e., THC, DHC, hemocyte viability) and humoral immune parameters (i.e., phenoloxidase-like activity, nitric oxide levels) in the hemolymph of *P. scaber* will have time dynamics following the LPS challenge that indicate transient or sustained immune responses. We discuss the importance of knowing the time-courses of such changes, to discriminate between transient responses that return the organism to its full function quickly, and sustained energetically costly immune activation. The results of this study help us to understand and interpret the outcomes of ecotoxicity studies, and serve as a basis for further research into the immunocompetence of such model organisms.

## Materials and Methods

### Experimental Organism

The terrestrial crustacean *Porcellio scaber* (woodlouse) was collected from a noncontaminated and pollution-free compost heap in Kamnik, Slovenia (46° 13′ 32.988″ N; 14° 36′ 42.12″ E). *P. scaber* were cultured at the University of Ljubljana (Slovenia) in glass containers under constant conditions of temperature (20 ± 2°C), high humidity, and photoperiod (16:8 h, light:dark) in a climate-controlled chamber. The glass containers were filled with soil from uncontaminated areas and leaf litter (*Corylus avellana, Alnus glutinosa*) was sterilized at 105°C for 3 h before being placed as a thick layer in the containers. Only healthy, adult woodlice (mean, 41.6 ± 0.6 mg fresh body mass) of both sexes were used. Molting individuals, females with marsupia, and woodlice showing symptoms of bacterial or viral infection (6) were excluded.

### Lipopolysaccharide Injection

For the injection of LPS, the *P. scaber* were divided into the control group (i.e., trauma control group) and the experimental group, each of which contained up to 35 individuals per time point. Body injections of *P. scaber* were performed between the 5th and 6th dorsal pereon segment using a 25-µL microsyringe (702N series, 33-gauge, blunt tip; Hamilton, Bonaduz, Switzerland) and a repeating dispenser (PB 600-1; Hamilton, Bonaduz, Switzerland), as described by Mayall et al. (20). The trauma control group was injected with 0.5 µL Dulbecco’s phosphate-buffered saline (DPBS; pH 7.1–7.5) and the experimental group was injected with the same volume of a sublethal dose of LPS (from *Escherichia coli* O111:B4; Sigma-Aldrich) dissolved in DPBS at a final concentration of 0.5 μg/μL. The LPS concentration was selected based on the results of a preliminary study (**Supplementary Figure 1**). The concentration that did not have any effect on the survival of the woodlice (mortality, <20%; 0.5 μg/μL) was chosen as the test concentration. At different time points after injection (i.e., 3, 6, 12, 24, 48 h postinjection), selected immune parameters were measured in the collected hemolymph from the trauma control and LPS challenged *P. scaber*. In addition, prior to the experiment (0 h), selected immune parameters were also measured in the hemolymph of *P. scaber* which had not been subjected to any challenge (culture control group; n = 35).

### Analysis of Cellular Immune Parameters

Total hemocyte count, DHC, and hemocyte viability were determined in duplicate for each hemolymph sample obtained from a single *P. scaber*, as described by Dolar et al. (6). Briefly, hemolymph was withdrawn by puncturing the intersegmental membrane with a sterile syringe on the dorsal site, between the 5th and 6th pereon segment. Using a glass capillary micropipette (Brand), the hemolymph was collected and immediately diluted at a ratio of 1:5 with DPBS and 0.4% trypan blue, which stains dead hemocytes, while the viable hemocytes remained unstained. Then 10 μL hemocyte suspension was pipetted onto a hemocytometer (Neubauer) to evaluate the THC, DHC, and hemocyte viability under light microscope (Axio Imager Z1; Zeiss). DHC was obtained from counting viable cells separately as semigranulocytes, granulocytes, and hyalinocytes using differential interference contrast microscopy technique. Degranulated semigranulocytes/granulocytes were not recognized and distinguished from each other, and therefore not included in DHC. In all, 10 hemolymph samples per group were analyzed for the cellular immune parameters.

### Analysis of Humoral Immune Parameters

Phenoloxidase-like activity in the hemolymph of the individual *P. scaber* (including plasma phenoloxidase and hemocyanine-derived phenoloxidase activities) was assessed photometrically, as described by Dolar et al. (6). Briefly, for *in-vitro* phenoloxidase activation, 3 μL of fresh hemolymph was diluted 1:39 with a solution containing DPBS buffer (pH 7.1–7.5), 8 mM dopamine hydrochloride, and 2 mM sodium dodecyl sulfate. Forty microliters of this 120-μL reaction mixture was transferred to 384-well plates. The formation of reddish-brown pigment was measured using Cytation 3 imaging reader (Biotek, USA), at 475 nm and 25°C, over at least 3 h. The phenoloxidase-like activity in the hemolymph was calculated as the change in absorbance from the linear part of the absorbance slope per min per μL hemolymph, multiplied by 10^3^, as described by Charles and Killian (37). In all, 10 hemolymph samples per group were analyzed for phenoloxidase-like activity.

Nitric oxide (NO) levels in the hemolymph were measured using Griess’ reagent, according to Dolar et al. (6). Briefly, 10 μL hemolymph was pooled from up to three *P. scaber* (in total 15 individuals per group), and added to 10 μL 100 mM potassium phosphate buffer (pH 7). The hemolymph suspension (20 μL) was mixed with 28 μL 1% sulfanilamide prepared in 5% H_3_PO_4_, and 48 μL 0.1% naphthylethylenediamine dihydrochloride, and kept on ice. The absorbance of NO was measured in 384-well plates at 543 nm after incubation for 5 min, using Cytation 3 imaging reader (Biotek, USA). NO concentrations (in μM) were calculated from a standard curve for NaNO_2_ (2.5–200 μM). In all, six hemolymph samples were analyzed per group for NO levels.

### Statistical Analysis

All statistical analyses and graphical representations were performed using the OriginPro v2021b software (OriginLab, Northampton, MA, USA). Data are presented as means ± standard error. For data meeting the assumptions of normality and homoscedasticity, two-sample t-tests were used, otherwise non-parametric Mann–Whitney U-tests were used. Significance was set at p <0.05. Pairwise comparisons between the culture control group and the trauma control groups, and between the trauma control group and the experimental groups, were performed for each time point.

## Results

### Total Hemocyte Count

**Figure 1:**
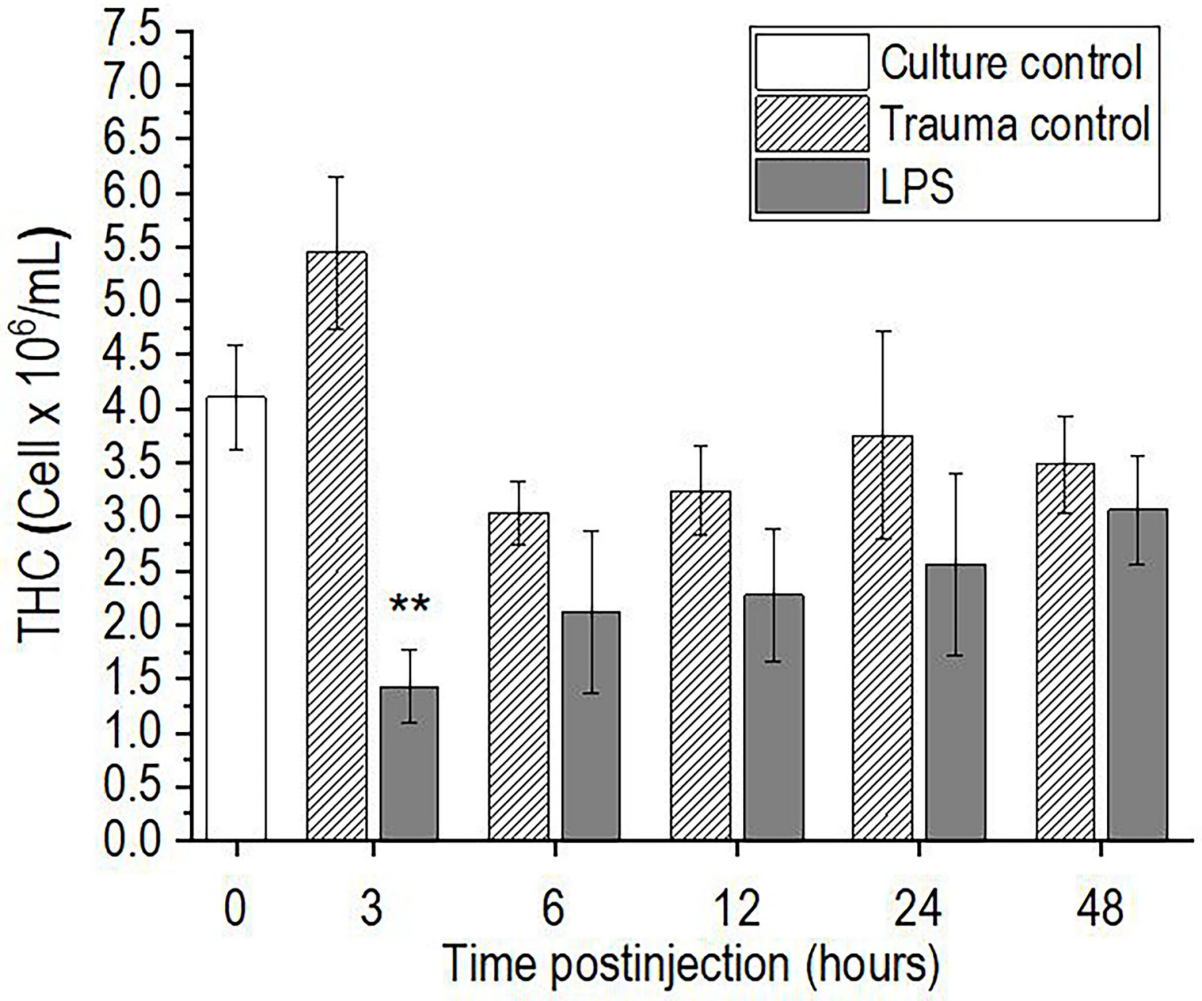
Time-course of total hemocyte count (THC) for the hemolymph of *P. scaber* injected with 0.5 μL DPBS (trauma control) and lipopolysaccharide (LPS; 0.5 μg/μL). Culture control represents *P. scaber* not subjected to any injection. Data are means ± SE. **p <0.01, *versus* trauma control (n = 10).

The injection of *P. scaber* with a single dose of 0.5 μg/μL LPS showed a statistically significant decrease in THC at the first time point of 3 h after stimulation, as compared to the trauma control (p <0.01) (**Figure 1**). This early decrease in THC after the LPS challenge was followed by return to initial values after 6 h, which then remained unchanged to the final time point of 48 h postinjection. In contrast, injection of an equal volume of DPBS buffer into *P. scaber* (trauma control) did not result in significant changes in hemocyte number compared to the culture control at any of the time points; however, a small increase in THC was observed 3 h after injection of DPBS buffer, although this did not reach significance compared to the culture control (**Figure 1**).

### Differential Hemocyte Count and Hemocyte Viability

**Figure 2:**
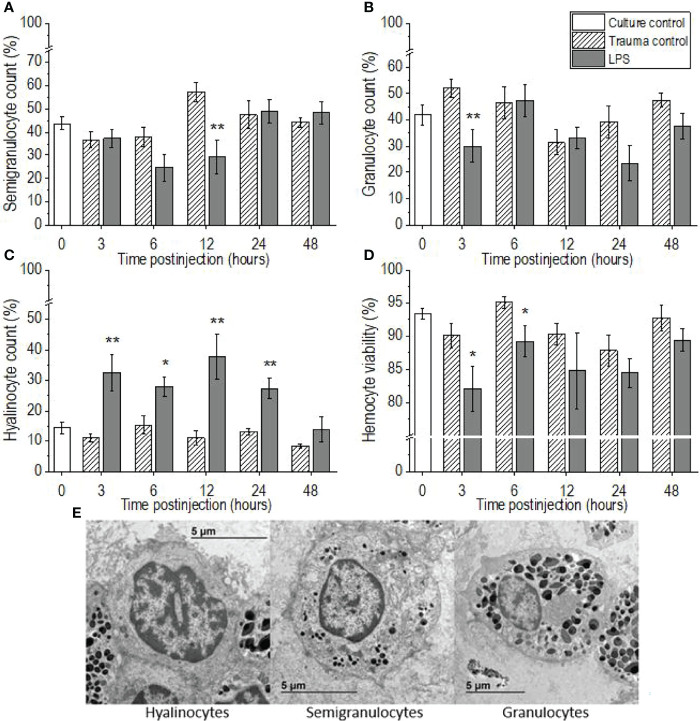
Time-courses of differential hemocyte counts **(A–C)** and hemocyte viabilities **(D)** for the hemolymph of *P. scaber* injected with 0.5 μL DPBS (trauma control) and lipopolysaccharide (LPS; 0.5 μg/μL). **(A–C)** Relative proportions of semigranulocytes **(A)**, granulocytes **(B)**, and hyalinocytes **(C)**. Culture control represents *P. scaber* not subjected to any injection. Data are means ± SE. *p <0.05; **p <0.01, *versus* trauma control (n = 10). **(E)** Transmission electron micrograph of the three main types of hemocytes (hyalinocytes, semigranulocytes, granulocytes) in the hemolymph of *P. scaber* (38).

Lipopolysaccharide challenge provoked significant changes in the proportions of the three hemocyte types in the hemolymph of *P. scaber*, compared to the injection of DPBS, which showed no significant changes compared to the culture control group at any of the time points (**Figures 2A**–**C, E**). The proportion of semigranulocytes in the hemolymph of the LPS-challenged *P. scaber* was decreased significantly at 12 h after injection, compared with the trauma control (p <0.01) (**Figure 2A**). Furthermore, a trend to a decrease in the proportion of semigranulocytes (p = 0.077) was observed 6 h after injection of LPS into *P. scaber*. Similarly, a significant decrease in the proportion of granulocytes was observed 3 h after injection of LPS (p <0.01) (**Figure 2B**). The proportion of hyalinocytes was changed the most, and these showed significant increases at 3 h (p <0.01), 6 h (p <0.05), 12 h (p <0.01), and 24 h (p <0.01) after injection of LPS, compared with the trauma control (**Figure 2C**). By 48 h after injection of *P. scaber* with LPS, the proportions of the hemocytes had recovered to the initial culture control values.

There were significant decreases in hemocyte viability in the hemolymph samples collected 3 h and 6 h after the LPS challenge, compared to the trauma control (p <0.05), whereas the injection of DPBS into the body of *P. scaber* (i.e., the trauma controls) caused no significant changes in hemocyte viability compared to the culture control (**Figure 2D**). However, by 48 h after injection of *P. scaber* with LPS, there were no differences in hemocyte viability between the control and experimental groups.

### Nitric Oxide Levels

**Figure 3:**
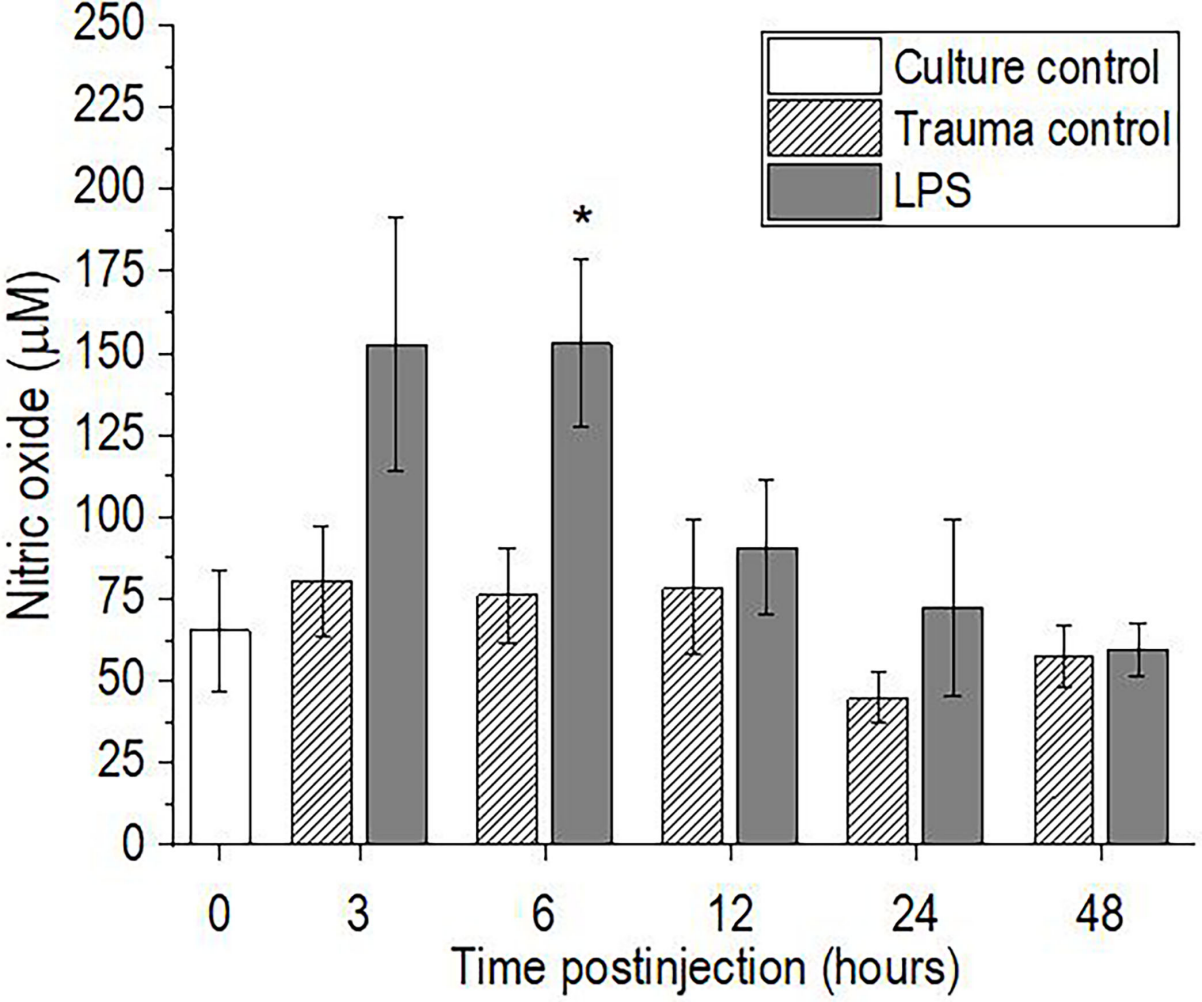
Time-course of nitric oxide levels for the hemolymph of *P. scaber* injected with 0.5 μL DPBS (trauma control) and lipopolysaccharide (LPS; 0.5 μg/μL). Culture control represents *P. scaber* not subjected to any injection. Data are means ± SE. *p <0.05, *versus* trauma control (n = 6).

The levels of NO in the hemolymph of *P. scaber* increased significantly 6 h after injection of LPS, compared to the trauma control (p <0.05), with a similar trend seen 3 h after the injection of LPS (p = 0.075) (**Figure 3**). Elevated NO levels subsequently returned to the initial culture control values at 12 h after injection of LPS, and remained unchanged to the final time point of 48 h. In contrast, injection of *P. scaber* with DPBS did not cause any changes in the levels of NO at any of the time points, compared to the culture control.

### Phenoloxidase-Like Activity

**Figure 4:**
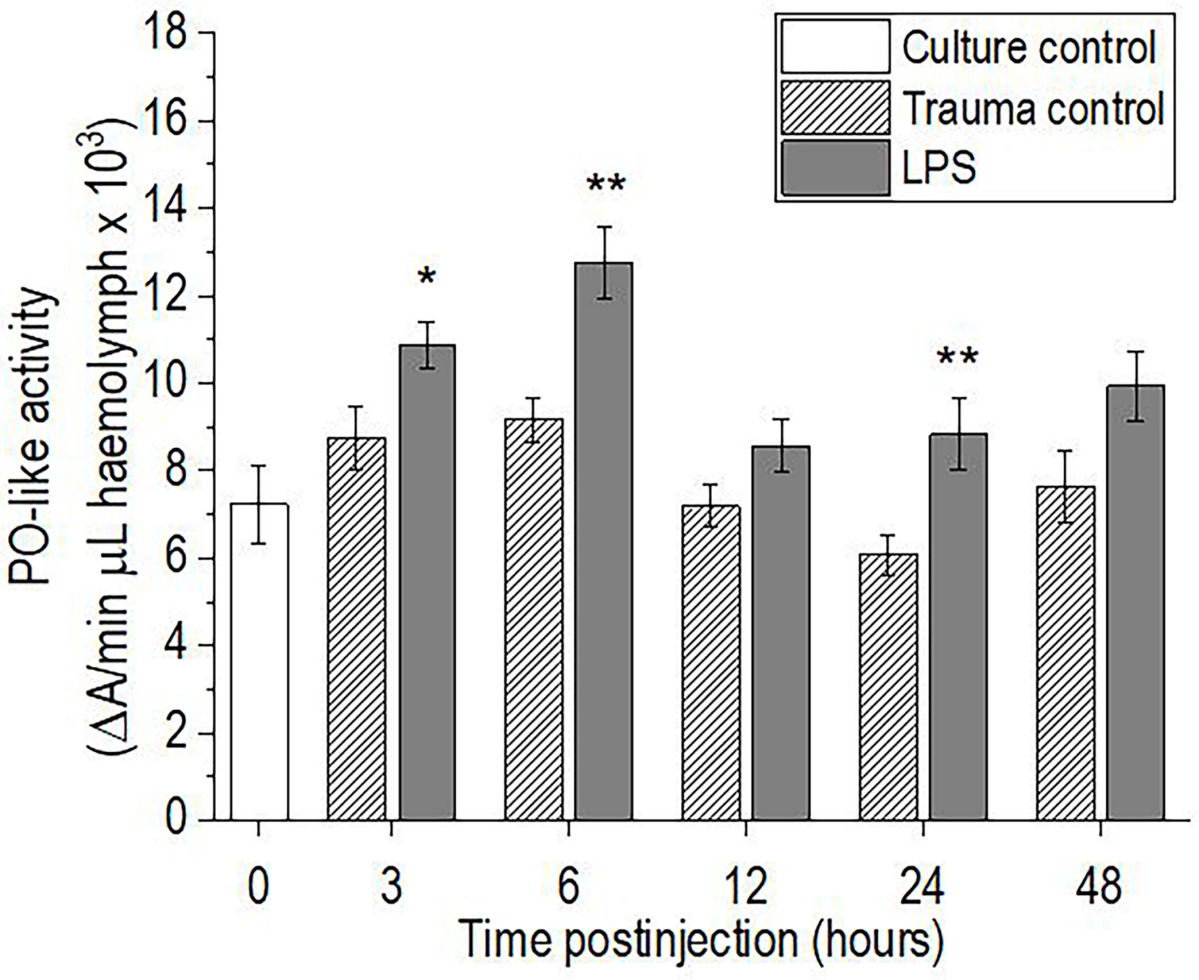
Time-course of phenoloxidase (PO)-like activity for the hemolymph of *P. scaber* injected with 0.5 μL DPBS (trauma control) and lipopolysaccharide (LPS; 0.5 μg/μL). Culture control represents *P. scaber* not subjected to any injection. Data are means ± SE. *p <0.05; **p <0.01, *versus* trauma control (n = 10).

Injection of *P. scaber* with LPS resulted in significant increases in hemolymph phenoloxidase-like activity at 3 h (p <0.05), 6 h (p <0.01), and 24 h (p <0.01) after the LPS challenge, compared with the trauma control. The hemolymph phenoloxidase-like activity then returned to the initial culture control levels by 48 h after injection with LPS (**Figure 4**). In contrast, injection of *P. scaber* with DPBS did not result in any significant changes in phenoloxidase-like activity at any of the time points, compared to the culture control.

## Discussion

This study examined the time-course of the innate immune response of the terrestrial crustacean *P. scaber* when challenged with LPS. The majority of the cellular and humoral immune parameters changed within 3-6 h, but then they all returned to the initial culture control levels already by 48 h after injection of LPS. It is known that the number of hemocytes changes dramatically after live bacteria and viruses or microbial polysaccharides such as LPS, laminarin, and β1,3-glucan (i.e., M/PAMPs) are introduced into the body of invertebrates (12–14, 22). This parameter has thus been used as a measure of stress and/or the physiological conditions of different species, including crustaceans (21, 37, 39, 40). In the present study, injection of LPS into the body of *P. scaber* resulted in a rapid decrease in the numbers of free hemocytes (by 3 h), followed by recovery to the baseline control levels 6 h after challenge. Many studies have reported such an early decrease in hemocyte numbers after M/PAMPs challenges in different crustaceans, which then gradually recover to the initial levels (12, 14, 26). For example, Ekblom et al. (14) and Lv et al. (12) explained the dramatic loss of hemocytes in the freshwater crayfish *Pacifastacus leniusculus* and in the Chinese mitten crab *Eriocheir sinensis* early after injection of live bacteria or M/PAMPs in terms of the migration of hemocytes from the hemocoel to the injection site, and the lysis of hemocytes, degranulation of granulocytes, coagulation, and nodulation or encapsulation of foreign particles. A similar decrease in THC after injection of LPS in the shrimp *Palaemon elegans* with a return of hemocyte number to initial level was also observed by Lorenzon et al. (41). This response is followed in the long term by the release of new cells from the hematopoietic tissue and their enhanced proliferation after a period of time following stimulation (42, 43), whereas a population of sessile hemocytes is responsible for the rapid regulation of hemocyte homeostasis (44). Therefore, the replenishment of circulating hemocytes (i.e., THC) to baseline levels observed in the present study (6 h after injection) can be explained by the rapid release of sessile hemocytes from the other tissue into the hemolymph. Another possible reason for the aforementioned initial decrease in hemocyte number in *P. scaber* could also be due to the known cytotoxicity of the LPS molecule, which impairs the viability of circulating hemocytes (16, 45). This is supported by our observations, showing decreased haemocyte viability at 3 h and 6 h after injection of LPS in *P. scaber*, which is consistent with the decrease in hemocyte numbers at 3 h after LPS injection. Since Xian et al. (16) reported that LPS challenge induce ROS/RNS overproduction, which is one of the major factors for hemocyte death in the shrimp *Penaeus monodon*, we explain the observed decrease in hemocyte viability in *P. scaber* after challenge with LPS as a result of LPS-induced oxidative damage.

In crustaceans, dead cells (including hemocytes) and also foreign particles, are removed by an evolutionarily conserved process, namely phagocytic activity for which hemocytes are responsible. This process is accompanied (among other effects) by excessive NO production in the hemocytes (46). Indeed, in our study we observed a trend towards an increase in NO levels in the hemolymph of *P. scaber* after 3 h; although this did not reach statistical significance, the effect was significant by 6 h after the LPS challenge. Similarly, in the crayfish *Procambarus clarkii*, a two-fold increase in NO levels was observed as early as 1 h after LPS injection, compared to those injected with saline (47). Moreover, Gopalakrishnan et al. (23) reported that in the crab *Scylla paramamosain*, LPS stimulation increased the levels of NO from 3 h to 96 h after the injection, while in addition phagocytic activity was also observed to be increased. The overproduction of ROS and RNS, which are responsible for degradation of phagocytosed particles, could have negative effects on the host cell viability. This was demonstrated in the current study and by Xian et al. (16), who reported that increased production of NO affected the apoptotic ratio of hemocytes in the shrimp *P. monodon* after LPS challenge. Another indication of increased phagocytic activity is also the increase in the proportion of hyalinocytes, which are generally responsible for phagocytosis of foreign particles and damaged cells (30, 36). However, these are indirect indications that phagocytosis was increased after LPS injection in *P. scaber*, as specific analysis of hemocyte phagocytic activity should be measured to confirm the occurrence of this process.

Injection of LPS into *P. scaber* did not cause aggregation of hemocytes, but it did alter the ratio of the three types of hemocytes in the hemolymph, with a marked increase in the proportion of hyalinocytes, which then recovered to the initial levels 48 h after injection of LPS. This is consistent with the observations of Xian et al. (48), who reported a similar increase 3 h to 24 h after LPS injection for the shrimp *P. monodon*, followed by a gradual recovery to the initial levels (after 48 h). Similarly, Zhou et al. (13) reported an increase in hyalinocyte numbers after stimulation of the crab *S. paramamosain* with the bacterium *Vibrio alginolyticus*, however the test organism was challenged with a suspension of live bacteria and not with microbial components, as was the case in our study. Furthermore, in the present study, a decrease in granulocyte and semigranulocyte proportions was observed 3 h and 12 h, respectively, after the LPS challenge. Similarly, Xian et al. (16, 48) reported significant decreases in the numbers of granular hemocytes (i.e., semigranulocytes, granulocytes) 3 h and 6 h after LPS challenge in the shrimp *P. monodon*, which was explained as a consequence of degranulation to release components of the proPO-activating system. Thus, the observed decrease in the proportions of granulocytes and semigranulocytes at 3 h, and at 6 h and 12 h, respectively, after LPS injection in *P. scaber* might be a consequence of hemocyte degranulation, followed by cell lysis and/or recruitment of cells (49–51). This explanation is also supported by the observations of Cardenas et al. (52) in the crayfish *Procambarus zonangulus*, and by Xian et al. (48, 53) in the shrimp *P. monodon* and *Litopenaeus vannamei*. However, it is difficult to distinguish between degranulated semigranulocytes and granulocytes, whereas these degranulated cells and hyalinocytes can be clearly distinguished based on differences in morphology. Another reason for the change in the proportions of the hemocyte types might also be the conversion between the main hemocyte types (e.g., granule accumulation or degranulation). For instance, some studies have indicated that the different types of hemocytes represent different stages of hemocyte development (54, 55).

Pathogen and parasite invasion and tissue damage are usually accompanied by melanization processes controlled by the enzyme phenoloxidase, whose activation is triggered by the presence of M/PAMPs or damage‐associated molecular pattern molecules (56, 57). In the case of LPS, it has been shown to specifically induce degranulation of semigranulocytes and granulocytes in crustaceans, leading to the release of components of the proPO system and its activation (49, 58, 59). Therefore, the observed increases in PO-like activity in the hemolymph of *P. scaber* at 3 h, 6 h, and 24 h after the LPS challenge can be explained as a response to LPS-associated cell and tissue damage. This is consistent with the observations made in other crustaceans, in which increased proPO gene expression and phenoloxidase activity have been observed following microbial infection (16, 23, 34), while some have also reported gradual recoveries of the phenoloxidase activity to baseline levels 24 h to 48 h after the challenge (16, 60, 61). A similar effect was also seen in the present study, where 48 h after LPS challenge of *P. scaber* the phenoloxidase-like activity returned to the initial culture control levels. This might also be because several toxic compounds (e.g., NO, which was increased in this study) are formed during melanization reactions that can harm the host (e.g., hemocyte viability), and therefore this process is tightly controlled (62, 63).

Overall, these data from the present study indicate that the response of *P. scaber* to injection with LPS at 0.25 μg LPS per woodlouse can be taken as a transient immune response. Thus, while this dose of LPS activated the innate immune system, the woodlice were able to recover by 48 h after the challenge. In contrast, in a study by Mayall et al. (20), some of the measured immune parameters in *P. scaber* were still strongly altered 48 h after exposure to a 100-fold higher dose of LPS (i.e., 25 μg LPS per woodlouse), which indicates a more sustained immune response due to the more severe LPS-induced damage (**Table 1**).

**Table 1:** Overview of the changes in the measured immune parameters in the hemolymph of *P. scaber* at different times after injection with a single dose of LPS (0.5 μg/μL).

Parameter	Time after LPS injection (h)					
	3	6	12	24	48	48^a^
Total hemocyte count	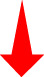 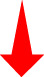	±	±	±	±	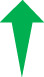 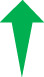
Hemocyte viability	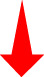	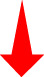	±	±	±	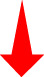 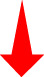
Semigranulocytes	±	±	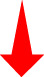 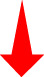	±	±	No data
Granulocytes	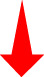 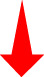	±	±	±	±	No data
Hyalinocytes	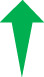 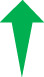	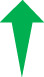	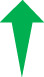 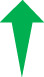	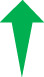 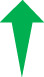	±	No data
NO levels	±	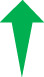	±	±	±	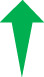 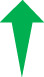
Phenoloxidase-like activity	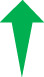	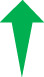 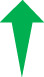	±	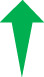 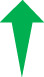	±	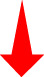

a Mayall et al. (20); data for P. scaber 48 h after injection with a higher dose of LPS (50 µg/µL).

^a^Arrows (↑/↓; ↑↑/↓↓) indicate statistically significant changes (p <0.05; p <0.01) in the measured immune parameters, while ± indicates no significant changes compared to the trauma control group.

The results presented here are particularly important in terms of arguing the general objectives of the use of immune responses in ecotoxicology. These are (a) to provide sensitive, rapid, and robust measures of stress responses; (b) to discriminate between transient and excessively prolonged, energetically costly immune activation; and (c) to determine whether a stressor can compromise the immunocompetence of an organism. We can conclude that the battery of humoral and cellular immune parameters used in the present study is suitable for identification of transient immune responses. In addition, we suggest that the injection of a low dose of LPS can be used to test the immunocompetence of an organism after exposure to other stressors, such as pollutants. For example, it has been demonstrated previously that *P. scaber* induces an immune response after exposure to certain pollutants, such as microplastics (7, 8); however, it remains to be determined whether these effects are transient or more sustained, or whether they also affect the immunocompetence. To answer these questions, pre-exposed organisms (e.g., to pollutants such as nano- and microplastics, pesticides) should be injected with LPS, for example, and humoral and cellular immune parameters should be measured after 48 h. This would indicate whether the immunocompetence of these pre-treated organisms was impaired compared to the nonpre-treated control organisms. We suggest that if the measured immune parameters differ in these two groups 48 h after stimulation with LPS, the pollutant exposure has affected their immunocompetence.

In conclusion, this study shows that LPS injection of *P. scaber* results in time-dependent innate immune responses, with dynamic and transient changes that are due to the expected cytotoxicity of LPS. As these immune parameters returned to their initial levels by 48 h after the LPS challenge, this indicates that the organism successfully handled the adverse effects of the LPS, i.e. the immune response was transient. Overall, these data confirm that these selected immune parameters are sensitive, rapid, and robust measures of the stress response. They can also be used to discriminate between transient and excessively prolonged, energetically costly immune activation. In addition, they can be used as tests to determine whether pre-exposure to a stressor impairs the immunocompetence of an organism.

## Data Availability Statement

The raw data supporting the conclusions of this article will be made available by the authors upon request, without undue reservation.

## Author Contributions

AD contributed to conception and design of the study. AD performed experiments, collected, and analyzed the data, and wrote the original manuscript. AD, DD, and AJ revised and edited the final manuscript. All authors contributed to the article and approved the submitted version.

## Funding

This research was funded by the Slovenian Research Agency (ARRS) through research programme Integrative Zoology and Speleobiology [grant number P1-0184]; research project J1-2482; and the ARRS funding scheme for postgraduate research for Andraž Dolar.

## Conflict of Interest

The authors declare that the research was conducted in the absence of any commercial or financial relationships that could be construed as a potential conflict of interest.

## Publisher’s Note

All claims expressed in this article are solely those of the authors and do not necessarily represent those of their affiliated organizations, or those of the publisher, the editors and the reviewers. Any product that may be evaluated in this article, or claim that may be made by its manufacturer, is not guaranteed or endorsed by the publisher.
