# Supplementary material for: Time-Course of the Innate Immune Response of the Terrestrial Crustacean Porcellio scaber After Injection of a Single Dose of Lipopolysaccharide
Source: Front Immunol. 2022 May 3;13:867077. doi: 10.3389/fimmu.2022.867077 (PMC9110979; doi:10.3389/fimmu.2022.867077)
Supplement: Supplementary file 1 [file DataSheet_1.docx]

Supplementary Material

# Results

**Supplementary Figure 1.** Survival of terrestrial crustacean *Porcellio scaber* 48 h after injection with 0.5 μL LPS at 0.5, 1.0, 5.0, 8.0 μg/μL, prepared in DPBS (pH 7.1-7.5). The same volume of DPBS was used as a trauma control.
